# A Treatise on Diseases of Infancy and Childhood

**Published:** 1872-07

**Authors:** 


### Books Reviewed.

*A Treatise on The Diseases of Infancy and Childhood.* Second Edition. Enlarged and thoroughly revised. By. J. LEWIS SMITH, M. D. Philadelphia: Henry C. Lea, 1872.

The Second Edition of Dr. Smith's work on diseases of children comes to us considerably enlarged and greatly improved. The first edition met with a very favorable reception at the hands of the profession and was lacking but few of the essentials of a complete text book of children's diseases. The additions and corrections which have been made in the second edition leave nothing to be desired to make a text book which as authority in this class of disease is second to none.

The manner in which the subject matter of the different chapters is treated shows the author to be familiar with this department of medicine in all its details, and gives evidence of that knowledge which can only be gained by experience. We advice our medical friends to purchase this book and give its contents their careful attention. All other departments of medicine are now undergoing, change and improvement. Careful perusal of this work will show that in the care of children, change and improvement is not less marked than elsewhere.

